# The protective effect of the TSPO ligands 2,4-Di-Cl-MGV-1, CB86, and CB204 against LPS-induced M1 pro-inflammatory activation of microglia

**DOI:** 10.1016/j.bbih.2020.100083

**Published:** 2020-05-21

**Authors:** Sheelu Monga, Nunzio Denora, Valentino Laquintana, Massimo Franco, Ilan Marek, Sukhdev Singh, Rafi Nagler, Abraham Weizman, Moshe Gavish

**Affiliations:** a Technion- Israel Institute of Technology, Ruth and Bruce Rappaport Faculty of Medicine, Israel; b Dipartimento di Farmacia – Scienze del Farmaco, Università degli Studi di Bari Aldo Moro, Italy; c Technion- Israel Institute of Technology, Schulich Faculty of Chemistry, Israel; d Sackler Faculty of Medicine, Tel Aviv University, Tel Aviv, Israel; e Research Unit, Geha Mental Health Center and Felsenstein Medical Research Center, Petah Tikva, Israel

**Keywords:** Translocator protein (TSPO), Neuroinflammation, Pro-inflammatory cytokines, Microglial activation, BV-2 ​cell line, M1 and M2 pathway, 2,4-Di-Cl-MGV-1, LPS (Lipopolysaccharide)

## Abstract

We have shown previously, that the 18 ​kDa translocator protein (TSPO) synthetic ligands quinazoline derivatives (2-Cl-MGV-1 and MGV-1) can inhibit activation of in BV-2 microglial cells. In the present study we assessed the impact of novel TSPO ligands on lipopolysaccharide (LPS)-induced microglial activation as expressed by release of pro-inflammatory molecules, including cytokines [interleukin-6 (IL-6), IL-1β, interferon- γ (IFN-γ)] nitric oxide (NO), CD8, and cyclo-oxygenase-2 (COX-2). The TSPO ligands 2,4-Di-Cl-MGV-1, CB86, and CB204 counteracted with the LPS-induced microglial activation. Exposure to LPS along with the TSPO ligand 2,4-Di-Cl-MGV-1 (25 ​μM) reduced significantly the release of NO by 24-, IL-6 by 14-, IL-β by 14-, IFN- γ by 6-, and TNF-α by 29-folds, respectively. In contrast to the anti-neuroinflammatory effect of the TSPO ligands, the effect of diclofenac sodium (DS; 25 ​μM) did not reach statistical significance. No alterations in IL-10 and IL-13 were detected (M2 anti-inflammatory pathway) during the inhibition of M1 pro-inflammatory pathway.

## 1 Introduction

Ligands active at the 18 ​kDa Translocator Protein (TSPO) exhibit immunomodulatory effects (Monga et al., 2019). TSPO is localized on the outer mitochondrial membrane and is involved in other biological functions such as: steroidogenesis, apoptosis, differentiation of neuronal progenitor cells, cholesterol transport, mitochondrial respiration, mitochondrial permeability transition pore opening, cellular proliferation, and modulation of cell nuclear gene expression (Gavish and Veenman, 2018; Veenman and Gavish, 2006). In our recent study, we have shown that the synthetic TSPO ligands 2-Cl-MGV-1 and MGV-1 appear to have beneficial effects in prevention of cell death and inflammatory processes in BV-2, a murine microglial cell line (Monga et al., 2019). Lately, we have synthesized another compound which is a derivative of 2-Cl-MGV-1 named 2,4-Di-Cl-MGV-1. In the present study, we analyzed the immunomodulatory properties of this ligand in BV-2 ​cell line. For the same study, we have also used another two TSPO ligands CB86 and CB204 (Denora et al., 2017; Iacobazzi et al., 2017).

The microglial cells are the brain’s resident macrophages and are involved in innate-inflammatory responses in the central nervous system (CNS) (Gan et al., 2017). M0, are the macrophages that are derived from monocytes that undergo terminal differentiation induced by the release of cytokines. M0 can be directed to a process of polarization depending on specific stimuli. The polarized macrophages inflammatory pathway can be classified into two major subtypes—pro-inflammatory M1 pathway and anti-inflammatory M2 pathway —based on the polarization concept of T-helper cells 1/T-helper cells 2 (Th1/Th2) (Koh et al., 2018).

Penetration of infectious agents via the blood brain barrier (BBB) into the brain, leads to microglial activation which eventually results in the secretion of pro-inflammatory cytokines including interleukin (IL)-1β, IL-6, tumor necrosis factor (TNF)-α, and interferon (IFN)-γ (Lue et al., 2001).

Microglial toll-like receptors (TLR)-4 are activated by exposure to lipopolysaccharide (LPS), an endotoxin produced by the cell wall of gram-negative bacteria (Lyman et al., 2014). The stimulation of TLR-4 by LPS leads to the activation of intracellular signaling mediators including NF-κB and MAPKs. The activation of this signaling cascades is associated with upregulation of pro-inflammatory molecules such as inducible nitric oxide synthase (iNOS), nicotinamide adenine dinucleotide phosphate (NADPH) oxidase, and cyclooxygenase 2 (COX-2), and subsequent release of pro-inflammatory cytokines (e.g. IL-1β, IL-6 and TNF-α), chemokines (e.g. MCP-1), nitric oxide (NO) and prostaglandins (PGs) (Monga et al., 2019; Choi et al., 2009). NO is generally unstable under aerobic conditions and oxidizes to its stable forms nitrite and nitrate (Pereira et al., 2013).

Activation of the NF-κB transcription factor leads to a marked macrophage-dependent immune response (Pang et al., 2015). These changes include the induction or suppression of a wide range of genes that regulate inflammation, cell proliferation, migration, and cell survival. Dyscontrol of this pathway can result in a rapid phosphorylation and subsequent ubiquitin dependent-degradation of the nuclear factor kappa in B-cells (IκB) α by the 26S proteasome (Majdalawieh and Ro, 2010).

Local effects of inflammation are usually beneficial, for example acute destruction of invading microorganism. It is also well known that chronic inflammatory response can have significant harmful effects (Fleit, 2014). Anti-inflammatory medications help in healing, prevention of more damage, and suppression of pain (Bortolasci et al., 2018). However, there is a need for better anti-inflammatory drugs, due to the side effects of non-steroidal anti-inflammatory drugs (NSAIDs) and recently, new synthetic TSPO ligands have been generated i.e. 2,4-Di-Cl-MGV-1, CB86 and CB204. Our previous studies have shown that the other two synthetic TSPO ligands 2-Cl-MGV-1 and MGV-1, can counteract with microglial activation and prevent pro-inflammatory responses up to 94% (Monga et al., 2019). We assessed the anti-inflammatory effects of 2,4-Di-Cl-MGV-1 (which is a derivative of 2-Cl-MGV-1) in BV-2 ​cells, a murine microglial cell line modelling primary microglial cell.

Microglia are pivotal players in innate immune/inflammatory responses in multiple neurologic disorders, including Alzheimer Disease (Mandrekar-Colucci and Landreth, 2010), Parkinson disease (Rogers et al., 2007), multiple sclerosis (Muzio et al., 2007), and amyotrophic lateral sclerosis (Dewil et al., 2007)(15). Activated microglia can secrete a wide range of inflammatory factors, including Th1 cytokines such as IL-1β, IL-6, TNF-α, and IFN-γ (Lue et al., 2001). TLRs have received particular attention with respect to their role in the injured CNS and possibly in Alzheimer Disease (Landreth and Reed-Geaghan, 2009). Stimulation of TLRs, of which there are at least 13 distinct members to date (Iwasaki and Medzhitov, 2004), induces activation of NF-kB and subsequent transcriptional activation of numerous pro-inflammatory genes (Nguyen et al., 2002).

In a recent study, we demonstrated the anti-inflammatory effects of the TSPO ligands 2-Cl-MGV-1 and MGV-1 (Monga et al., 2019; Azrad et al., 2019).

The aim of the present study was to evaluate the anti-inflammatory activity of the novel TSPO ligands 2,4-Di-Cl-MGV-1, CB86, and CB204 in BV-2 microglial cell line activated by LPS. To this end we assessed the inhibitory effect of the ligands on LPS-induced inflammatory response in BV-2 ​cells, as reflected by cell viability, metabolic activity, nitric oxide (NO) production, release of pro-inflammatory cytokines [interleukin-6 (IL-6), IL-1β, interferon- γ (IFN-γ)], and the expression of CD8, cyclo-oxygenase-2 (COX-2) and NF-κB p65. Diclofenac sodium (DS; 25 ​μM) was used as an anti-inflammatory comparator. We expected that these ligands would exhibit better anti-inflammatory activity compared to the previously studied two TSPO ligands (2-Cl-MGV-1 and MGV-1) due to a better affinity to TSPO.

## 2 Methods

### 2.1 -(2,4-dichlorophenyl)quinazolin-4-yl dimethylcarbamate (2,4-Di-Cl-MGV-1)

**Figure:**
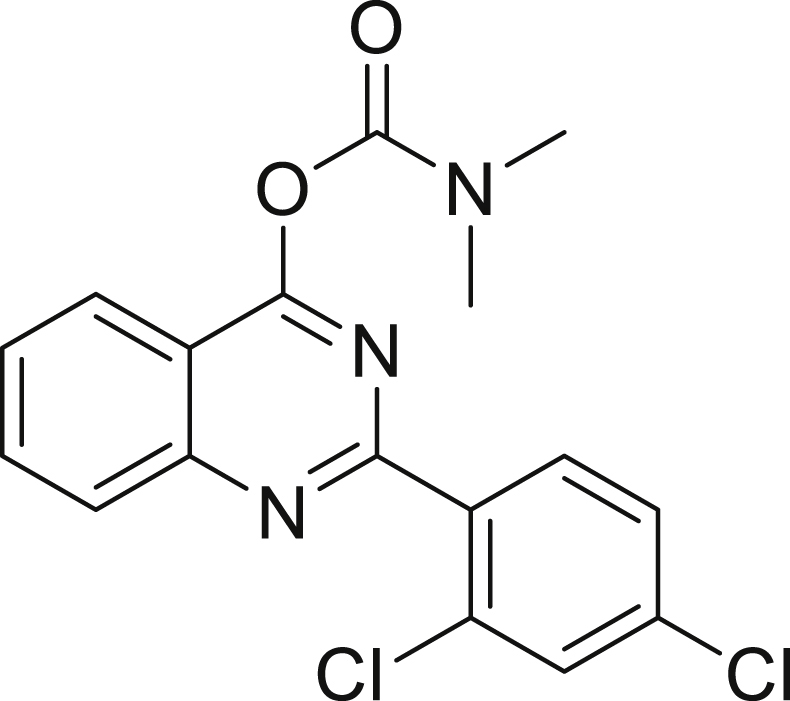

### 2.2 Synthesis of 2,4-Di-Cl-MGV-1

**Figure:**
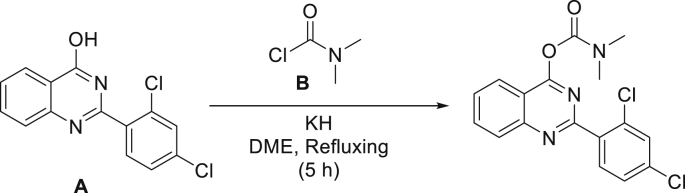

**Procedure:** To a suspension of potassium hydride (6.75 ​mmol) under argon in 30 ​ml of 1,2-dimethoxyethane (DME) is added the 2-(2,4-dichlorophenyl)quinazolin-4-ol (A) (4.5 ​mmol) in one portion. The reaction mixture was stirred for 30 ​min at room temperature and the carbamoyl chloride (B) (7 ​mmol, dissolved in 10 ​mL of DME) was added dropwise to the reaction mixture. Reaction mixture was then brought to reflux for 5 ​h till completion of reaction (TLC monitored). The reaction mixture was then quenched with 30 ​mL of water carefully, followed by extraction with dichloromethane (3 ​× ​50 mL). Combined organic phases were dried over MgSO4 and the solvents were evaporated under reduced pressure. The resulting crude product was purified by silica gel chromatography using hexane/EtOAc as eluent (85/15).

### 2.3 CB86 and CB204

The two compounds CB86 and CB204 were synthesized and apparently, the affinity of CB86 is better than the classical TSPO ligand PK 11195 (Denora et al., 2017; Iacobazzi et al., 2017).

**Figure:**
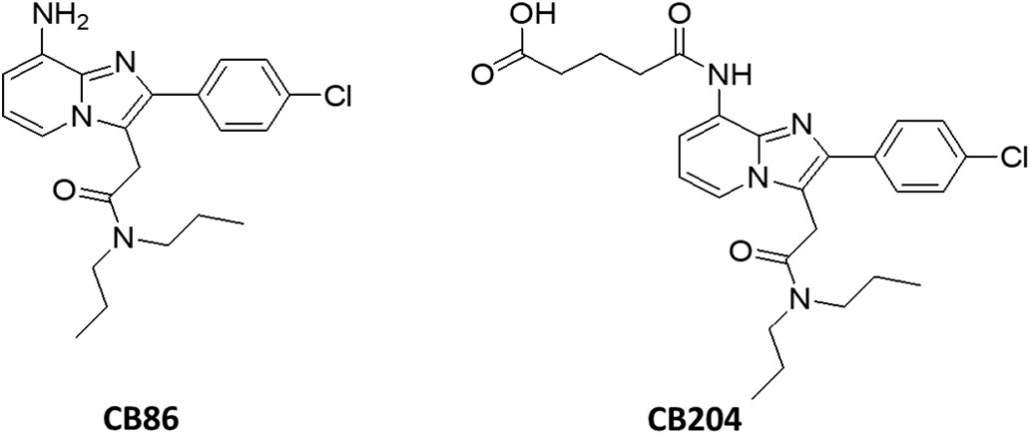

### 2.4 BV-2 ​cells

We used an *in-vitro* model of M1 inflammatory pathway using murine BV-2 ​cells, derived from raf/myc-immortalized murine neonatal microglia (a gift from Professor Zvi Vogel laboratory, Weizmann Institute of Science). These cells were found to be appropriate for pharmacological characterization, including immunological functions. These cells can be activated by LPS or IFN-γ (Henn et al., 2009).

These BV-2 microglia cells were cultured at 37 ​°C in an atmosphere of 5% CO_2_ and 90% relative humidity. The BV-2 ​cells were incubated in Dulbecco’s modified Eagle’s medium containing 4.5 ​g/L glucose and 4 ​mM L-glutamine and supplemented with 5% fetal bovine serum, penicillin (100 U/ml), and streptomycin (100 ​μg/ml) (Bae et al., 2014).

### 2.5 Lipopolysaccharide (LPS) exposure

BV-2 ​cells were seeded in 6-wells plate. After 48 ​h, the cells were exposed to 100 ​ng/ml LPS (Sigma-Aldrich, Rehovot, Israel) for 24 ​h, as previously described (Monga et al., 2019). Medium was collected and cells were scrapped off for the required assays following the exposure to LPS. The confluency of BV-2 ​cells at the time of treatment was 80%.

### 2.6 Treatments with the TSPO ligands and DS

**Figure 1:**
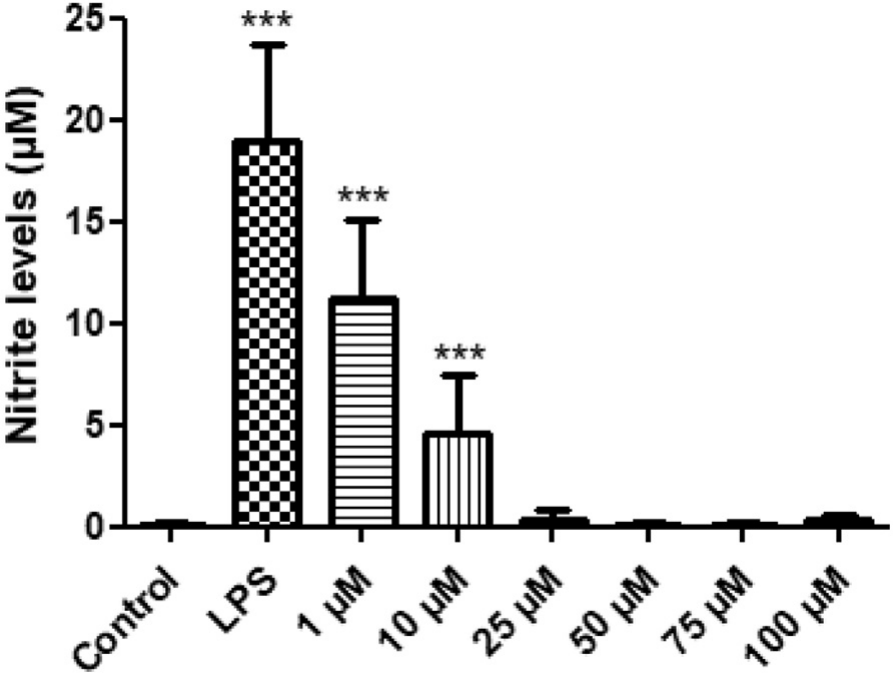
**2,4-Di-Cl-MGV-1 dose response with nitrite assay along with LPS exposure.** Concentration-dependent effect of 2,4-Di-Cl-MGV-1 against the inflammatory effect of LPS. ANOVA with Bonferroni’s post-hoc test was performed. ∗∗∗p ​< ​0.001 compared to control group.

Treatments included: 24-h exposure of BV-2 ​cells to LPS (100 ​ng/ml), with or without the treatment of 2,4-Di-Cl-MGV-1 and DS (25 ​μM of each). In a dose-response experiment of 2,4-Di-Cl-MGV-1, it was shown that already at 1 ​μM 2,4-Di-Cl-MGV-1 has a significant inhibitory effect on microglial activation but, the effect of 10 ​μM of 2,4-Di-Cl-MGV-1 was significantly higher than control. The 25 ​μM concentration was found to be optimal for restoration of the inflammatory markers to control levels (Fig. 1). Therefore, we chose this concentration in all our experiments.

### 2.7 Trypan blue exclusion dye assay

This method was used to count the viable cells in order to seed a same number of cells for the various groups of each experiment. Viable cells exclude the Trypan blue dye, whereas nonviable cells absorb the dye and appear blue. The assay was performed as previously described (Monga et al., 2019; Veenman et al., 2004). Briefly, cells were scrapped off, and were re-suspended in fresh medium and a 200 ​μl sample was collected for cell counting. Cells were stained with Trypan blue (200 ​μl) at a final concentration of 0.05% and counted by hemocytometer. Cell counting was performed using an inverted microscope.

### 2.8 Metabolism assay with XTT

We used the cell metabolic-XTT-based assay kit (Biological Industries, Beit HaEmek, Israel), to assess the metabolic activity of BV-2 ​cells exposed to LPS. According to the instructions of the manufacturer, briefly, the 2,3-bis [2-methoxy-4-nitro-5-sulphophenyl]-2H-tetrazolium-5-carboxyanilide inner salt (XTT) assay is based on reduction of XTT by mitochondrial dehydrogenases of viable cells yielding an orange formazan product. Absorbance at 492 ​nm with reference at 620 ​nm was measured with the Spectrophotometer Zenyth 200 (Anthos, Eugendorf, Austria) (Monga et al., 2019).

### 2.9 Nitrite assay

Nitrite and nitrate are the two stable forms of nitric oxide (NO) which are released during inflammatory responses by iNOS. BV-2 ​cells were seeded in 24 well plate and incubated for 72 ​h in complete medium. Then, the cells were exposed to 100 ​ng/ml of LPS and treated with 2,4-Di-Cl-MGV-1 or DS (25 ​μM). Nitrite production was determined by measuring the levels of the NO metabolite nitrite (NO2) in the medium by using a colorimetric reaction with Griess reagent (explained in Monga et al., 2019). Then equal volumes of cell culture supernatant and Griess reagent were mixed (100 ​μL). sodium nitrate, 0.1 ​M was used to build a calibration curve. Absorbance was measured at 540 ​nm after 15 ​min on a shaker, with a Spectrophotometer Zenyth 200 (Anthos, Eugendorf, Austria).

### 2.10 Enzyme-linked immunosorbent assay (ELISA)

ELISA kits were used for the assessment of the expression of IL-1β (ab197742), TNF-α (ab208348), IFN-γ (ab100689), IL-6 (ab222503), IL-10 (ab108870), and IL-13 (ab219634) in the cell culture supernatant and NF-κB p65 (ab176647) and COX-2 (ab210574) in cell lysates [Abcam, Zotal Ltd., Tel Aviv, Israel]. All the detected markers were compared between LPS-exposed cells and LPS ​+ ​ligands-treated cells. All the reagents provided in the kit were ready for use and stored according to the manufacturer’s instructions.

Sample preparation: Cell culture supernatants were collected and centrifuged at 210 ​g to remove floating cells. Cell culture supernatants were carefully collected, aliquoted and stored in −20 ​°C. For lysate samples, growth medium was removed by aspiration with vacuum, and cells were washed 2 times in PBS. Cells were scraped off and resuspended by the addition of chilled 1X Cell Extraction Buffer PTR (to lyse the cells with 100 ​μL of 1X Cell Extraction Buffer PTR). Cells were incubated on ice for 20 ​min and then centrifuged at 15,300 ​g for 5 ​min at 4 ​°C. Supernatant was collected, aliquoted, and stored in −80 ​°C immediately.

Plate preparation and standards: The kits (all the above-mentioned ELISA kits) provided ready to use plates and desired number of wells were used for the experiment. 50 ​μL of all samples, controls and standard were added to the appropriate wells. 50 ​μL of the antibody cocktail was added to the samples in each well. The wells were sealed and incubated for 1 ​h at room temperature on a plate shaker set to 400 ​rpm. Plate was washed with 3 ​× ​350 ​μL 1X Wash Buffer PT. TMB, 100 ​μL substrate was added and incubated for 15 ​min in the dark on a plate shaker set to 400 ​rpm 100 ​μL of stop solution was added and mixed on a shaker for 1 ​min to stop the reaction. O.D. was recorded the at 450 ​nm with endpoint reading.

### 2.11 Preparation of kidney membranes

Rat kidney membranes were used in the binding assays since it expresses the TSPO and is an established tissue for the binding characteristics of TSPO. Sprague Dawley rats were anesthetized with ketamine (87.5 ​mg/kg) and xylazine (12.5 ​mg/kg) cocktail that was injected IP. The rats were sacrificed by decapitation. The kidneys were collected and homogenized for 30 ​s using a Brinkmann Polytron (setting 10) in 50 ​mM phosphate buffer, pH 7.4. The homogenate was centrifuged at 49,000×*g* at 4 ​°C for 20 ​min. The pellet was resuspended in around 50 ​ml of 50 ​mM ice cold phosphate buffer, pH 7.4 to achieve 750 ​μg/ml protein. The protein concentration was determined using Bradford assay.

### 2.12 [^3^H] PK 11195 binding to kidney membrane

The ability of unlabeled PK 11195, MGV-1, 2-Cl-MGV-1, and 2,4-Di-Cl-MGV-1 to compete with [^3^H] PK 11195 (2 ​nM final concentration) binding to kidney membranes was assessed. The binding assay in final volume of 500 ​μL containing 25 ​μL of [^3^H] PK 11195 (2 ​nM final concentration) in the absence (total binding) or the presence of various concentrations of unlabeled ligands (1 ​nM–10 ​μM, final concentration) and 400 ​μL of kidney membranes (300 ​μg protein). The mixture was incubated for 90 ​min on ice. Samples were filtered through Whattman GFC filters under vacuum and washed three times with 50 ​mM phosphate buffer. Filters were placed in vials containing 3 ​ml of liquid scintillation (LSC) cocktail. The samples were counted for radioactivity after 12 ​h.

### 2.13 Statistical analysis

Results are presented as Mean ​± ​standard deviation (SD). Two-tailed Wilcoxon, Student’ t and one-way analysis of variance (ANOVA) tests were used as appropriate, including Bonferroni’s post-hoc test. Statistical significance was defined by p ​< ​0.05.

## 3 Results

We compared the ability of unlabeled PK 11195, MGV-1, 2-Cl-MGV-1, and 2,4-Di-Cl-MGV-1 to compete with [^3^H] PK 11195 (2 ​nM final concentration) binding to kidney membranes (Table 1). Usually the kidney membranes are used to compare the affinity of various ligands to TSPO. In this study, we compared the affinities of the three synthetic TSPO ligands to the classical TSPO ligand PK11195. The results present the average of 3 separate experiments conducted in triplicates with less than 10% variability (Table 1).

**Table 1:** **IC**_**50**_**values of TSPO ligands using [**^**3**^**H] PK 11195 (2 ​nM final concentration) binding to rat kidney membranes** at 4 ​°C for 90 ​min in the absence (total binding) or the presence of unlabeled PK 11195, MGV-1, 2-Cl-MGV-1 and 2,4-Di-Cl-MGV-1 at a concentrations ranging from 1 ​nM to 10 ​μM.

TSPO ligands	IC_50_values (nM)
PK 11195	6
MGV-1	350
2-Cl-MGV-1	350
2,4-Di-Cl-MGV-1	100
CB86	1.6
CB204	117.7

### 3.1 2,4-Di-Cl-MGV-1 dose response

1% ethanol has been used as a vehicle in this study as there was no impact of the 1% ethanol on the viability of the BV-2 ​cells according to Monga et al., 2019) (Monga et al., 2019). We have also assessed the 2,4-Di-Cl-MGV-1 dose response to check the pro-inflammatory inhibition induced by 100 ​ng/ml LPS. Already at 1 ​μM concentration of 2,4-Di-Cl-MGV-1, significantly inhibited the pro-inflammatory response in nitrite assay (Fig. 1).

### 3.2 Competitive study of 2,4-Di-Cl-MGV-1, CB86, CB204, and DS with nitrite assay

**Fig. 2:**
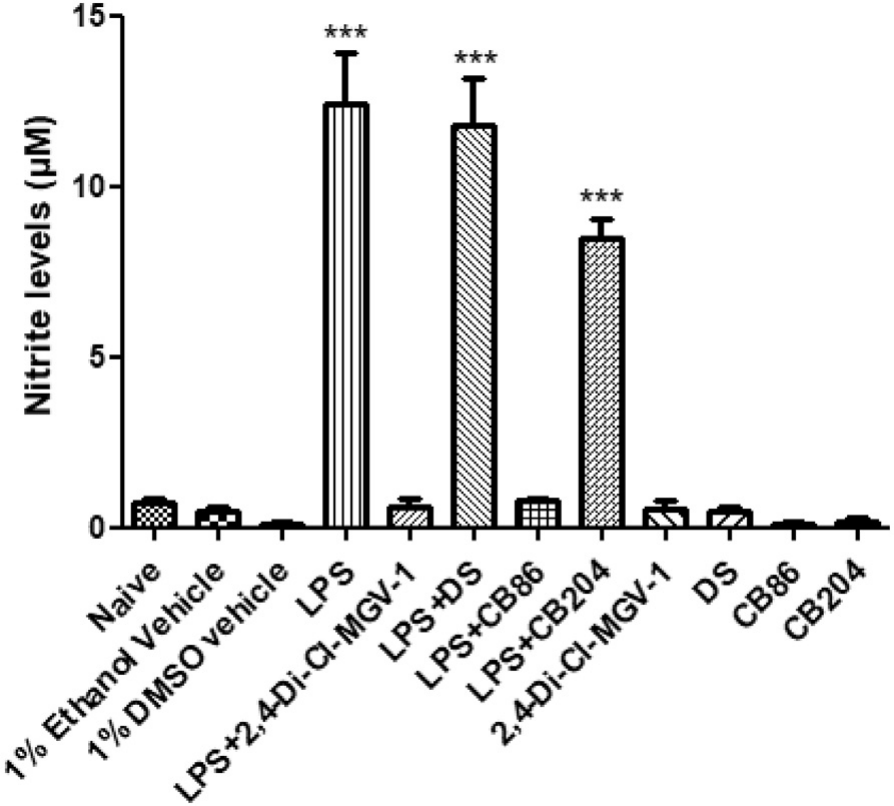
**Comparative study of TSPO ligands 2,4-Di-Cl-MGV-1, CB86, CB204 and DS on nitrite levels.** BV-2 microglial cell line was exposed to 100 the ng/ml LPS for 24 ​h in the presence or absence of TSPO ligands 2,4-Di-Cl-MGV-1, CB86, CB204, and NSAID DS (25 ​μM each). Nitrite levels (μM) were calculated using a standard calibration curve and are presented as means ​± ​SD; n ​= ​8 for naïve, 1% ethanol vehicle, LPS+2,4-Di-Cl-MGV-1, LPS ​+ ​DS, 2,4-Di-Cl-MGV-1, and DS; n ​= ​4 for 1% DMSO vehicle, LPS ​+ ​CB86, LPS ​+ ​CB204, CB86, and CB204; n ​= ​12 for LPS group. ANOVA followed by Bonferroni’s post-hoc test was performed, and the results remained statistically significant ∗∗∗ compared to all other groups (p ​< ​0.001 for all).

Nitric oxide (NO) is one of the inflammatory markers but under aerobic conditions, it spontaneously oxidizes to its stable forms i.e. nitrite and nitrate. So, nitrite levels were analyzed as an inflammatory marker. BV-2 ​cells were exposed to 100 ​ng/ml of LPS along with 25 ​μM of 2,4-Di-Cl-MGV-1, CB86, CB204 or DS for 24 ​h. Nitrite level in the LPS group was dramatically elevated but in the case of LPS+2,4-Di-Cl-MGV-1 and LPS ​+ ​CB86, induction of nitrite was prevented completely (around 24-folds). In contrast, DS did not prevent significantly the induction of nitrite by LPS and CB204 has some effect for the inhibition of nitrite levels, but it is not as good as 2,4-Di-Cl-MGV-1 and CB86 (Fig. 2). All the TSPO ligands and DS did not affect nitrite levels on their own.

Also, there was no significant difference between naïve, 1% ethanol vehicle and 1% DMSO vehicle. Thus, we used 1% ethanol in the case of 2,4-Di-Cl-MGV-1 and DS; whereas 1% DMSO as a control in the case of CB86 and CB204.

### 3.3 Assessment of the levels of pro-inflammatory cytokines by ELISA

**Fig. 3:**
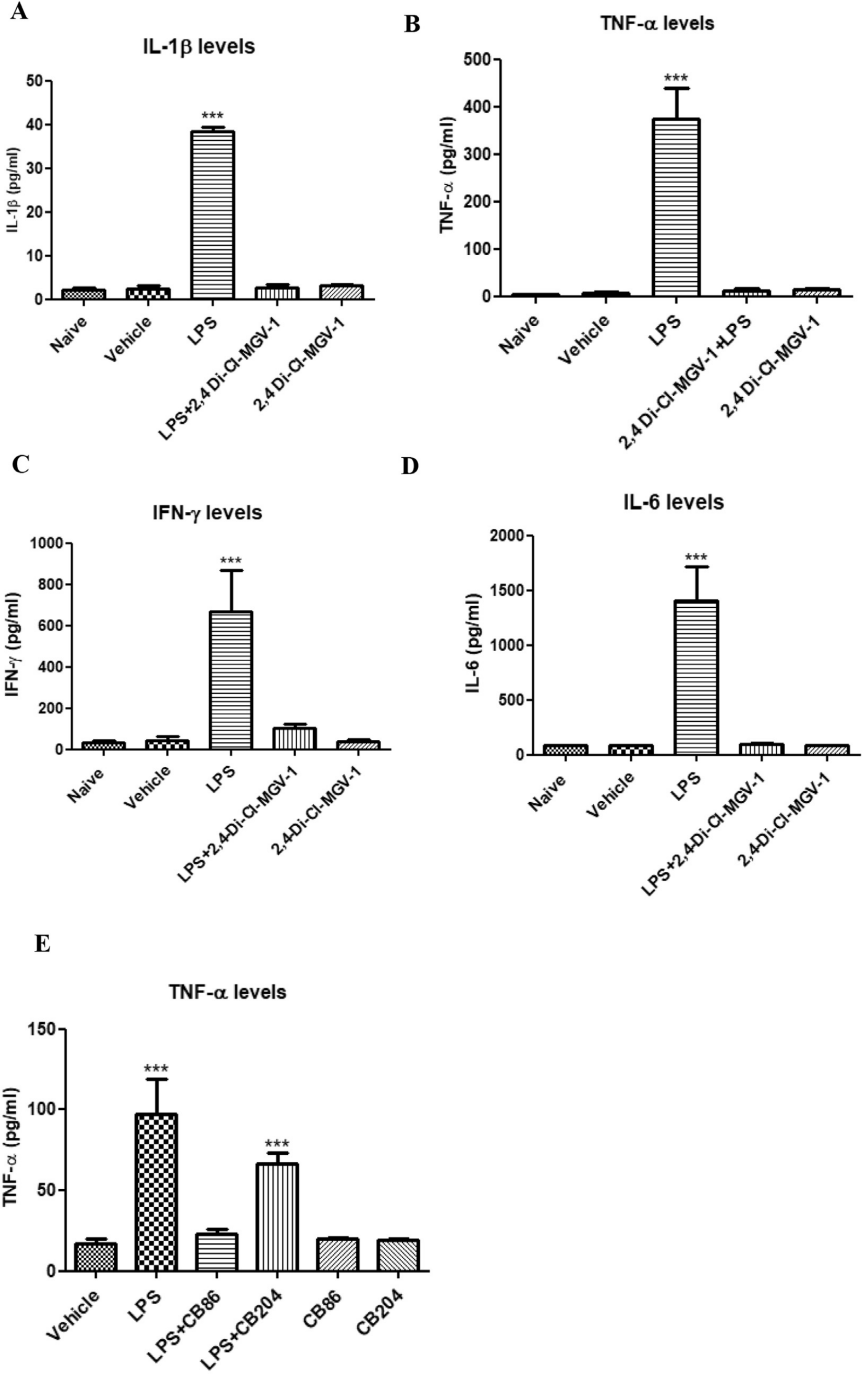
**Effect of TSPO ligands on pro-inflammatory cytokines**. BV-2 ​cells were exposed to TSPO ligands (25 ​μM) simultaneously with 100 ​ng/ml of LPS for 24 ​h. (A) IL-1β; (B) TNF-α; (C) IFN-γ; (D) IL-6 and (E) TNF-α (for CB86 and CB204) concentrations (pg/ml) were calculated using a standard calibration curve and are presented as Means ​± ​SD; 4 replicates in each group. ANOVA with Bonferroni’s post-hoc test was performed. ∗∗∗p ​< ​0.001 compared to all other groups.

The effects of 2,4-Di-Cl-MGV-1 on IL-1β, TNF-α, IFN-γ, and IL-6 generation as a part of pro-inflammatory response of BV-2 ​cells induced by LPS were assessed using ELISA (Fig. 3A–D). No difference between naïve and vehicle control groups were detected at baseline. In comparison to naïve and the vehicle groups, IL-1β, TNF-α, IFN-γ, and IL-6 generation in the LPS groups were dramatically increased. In this paradigm, 2,4-Di-Cl-MGV-1 successfully inhibited all the pro-inflammatory cytokine generation as compared to naïve/vehicle levels (IL-6 by 14-folds; IL-1β by 14-folds; IFN- γ by 6-folds; and TNF-α by 29-folds). By itself, 2,4-Di-Cl-MGV-1 did not affect the levels of the pro-inflammatory cytokines. Fig. 3E shows that the effect of TSPO ligands CB86 and CB204 against the LPS-induced microglial activation of TNF-α release. CB86 effectively inhibited the LPS-induced TNF-α levels while CB204 was i efficient enough (4.2-folds and 1.4-fold, respectively) with respect to the inflammatory responses. Also, these ligands did not have such an effect on their own.

### 3.4 NF-κB p65 (pS536) ELISA assay

**Fig. 4:**
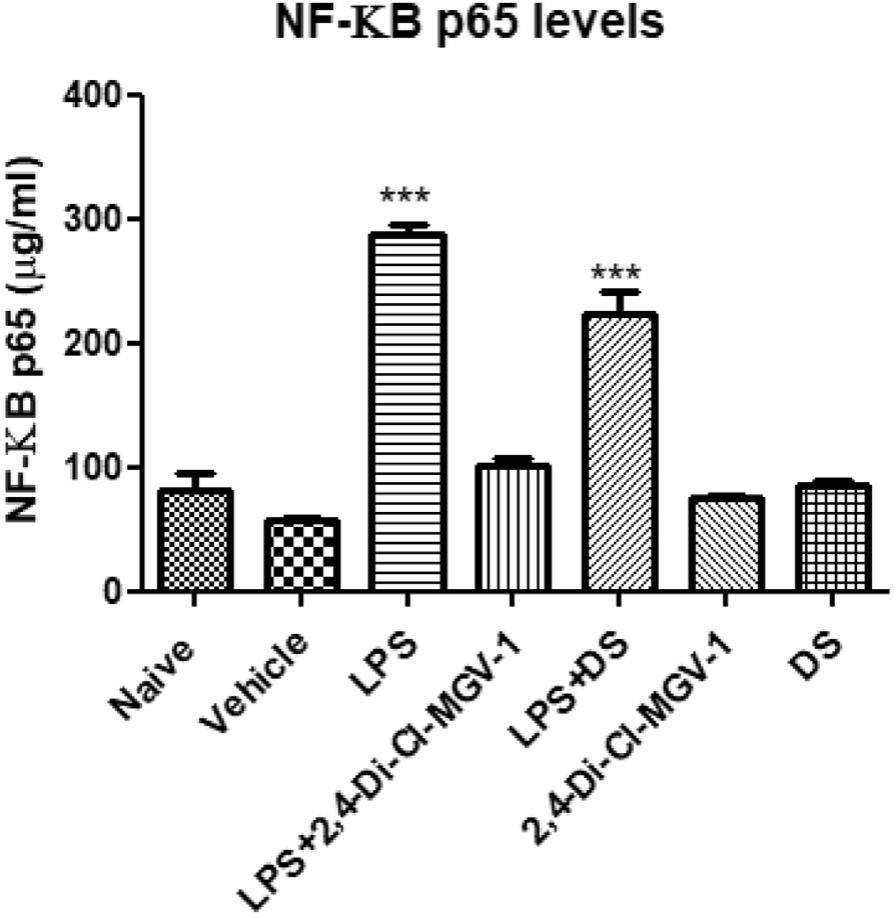
**Effect of the 2,4-Di-Cl-MGV-1 on NF-κB p65 (pS536) levels (measured by ELISA) in LPS- stimulated BV-2 ​cells**. BV-2 ​cells were exposed to 100 ​ng/ml LPS for 24 ​h with or without 2,4-Di-Cl-MGV-1 or DS (25 ​μM each). Concentrations (μg/ml) were calculated using a standard calibration curve and are presented as Means ​± ​SD; 4 replicates in each group. ANOVA with Bonferroni’s post-hoc test was performed (p ​< ​0.001 compared to all other groups).

We conducted an NF-κB p65 (pS536) ELISA assay in order to detect NF-κB p65 activation. As seen in Fig. 4, the LPS compared to the vehicle group expressed 5-fold increase in NF-κB p65. Treatment with 2,4-Di-Cl-MGV-1 decreased significantly the NF-κB p65 content to the control range. Co-treatment of LPS with DS resulted in a decrease of 30% of NF- κB p65 content. However, these values remained significantly higher than naïve and vehicle levels.

### 3.5 COX-2 assay with ELISA

**Fig. 5:**
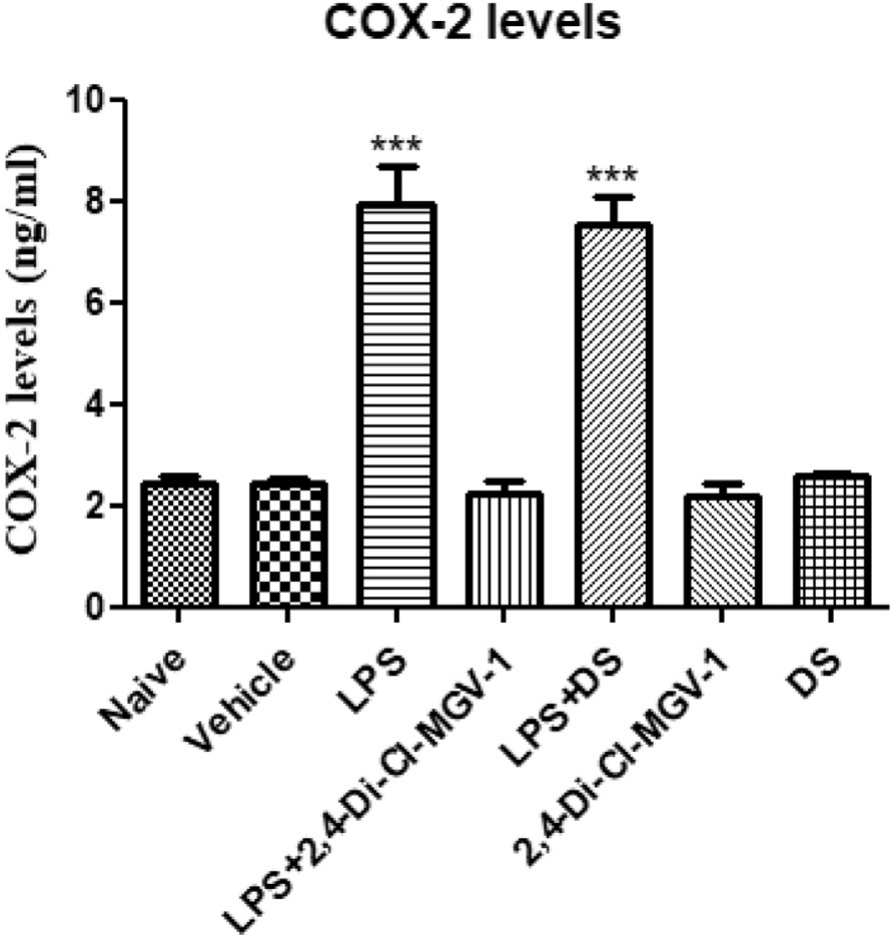
**Effect of the 2,4-Di-Cl-MGV-1 on COX-2 levels (measured by ELISA) in LPS- stimulated BV-2 ​cells**. BV-2 ​cells were exposed to 100 ​ng/ml LPS for 24 ​h with or without 2,4-Di-Cl-MGV-1 and DS (25 ​μM each). Concentrations (ng/ml) were calculated using a standard calibration curve and are presented as Means ​± ​SD; 4 replicates in each group. ANOVA with Bonferroni’s post-hoc test was performed (p ​< ​0.001 compared to all other groups).

COX-2 is an intracellular marker of the inflammatory pathway. Therefore, we conducted COX-2 ELISA assay. As shown in Fig. 5, LPS group expressed more than 3-fold increase in COX-2 levels as compared to vehicle. Treatment with 2,4-Di-Cl-MGV-1 decreased significantly the COX-2 levels to the control range. DS also has a small non-significant reduction of only 10% as compared LPS groups.

### 3.6 CD8 levels with ELISA

**Fig. 6:**
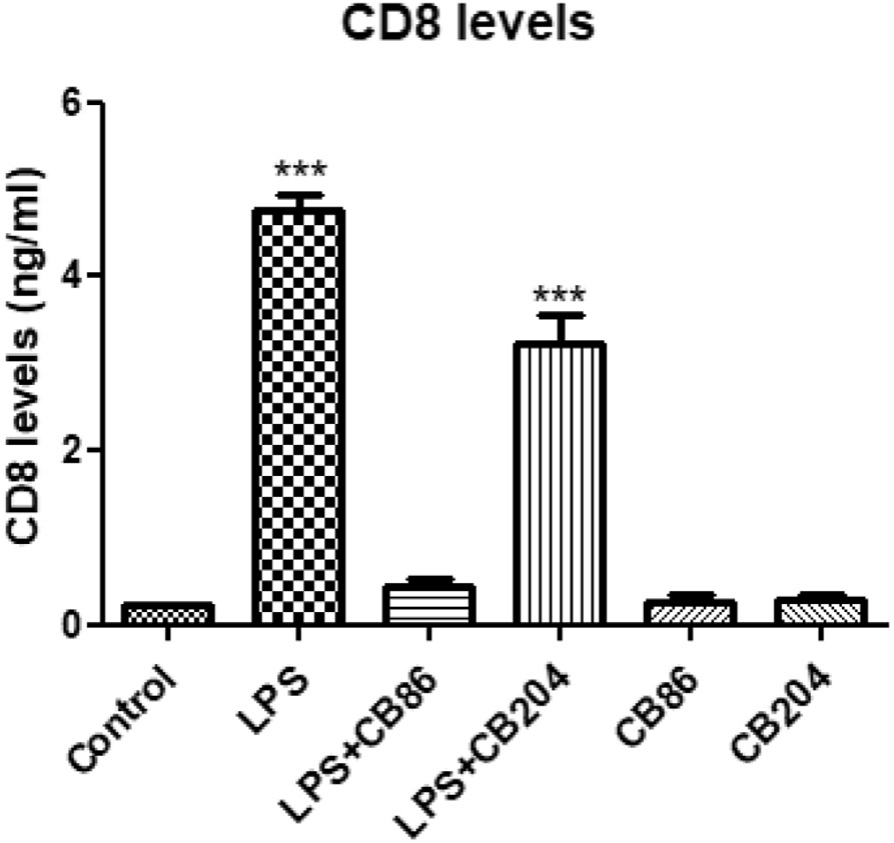
**Effect of the two TSPO ligands CB86 and CB204 on CD8 levels in LPS- stimulated BV-2 ​cells.** BV-2 ​cells were exposed to 100 ​ng/mL LPS for 24 ​h with or without the two TSPO ligands (25 ​μM each). CD8 levels (ng/mL) were calculated using a standard calibration curve and are presented as Means ​± ​SD; n ​= ​4 in each group. ANOVA followed by Bonferroni’s post-hoc test was performed, and the results remained statistically significant ∗∗∗p ​< ​0.001 compared to all other groups.

CD8 is a transmembrane glycoprotein that serves as a co-receptor for the T cell receptor (TCR). Like the TCR, CD8 binds to a major histocompatibility complex (MHC) molecule but is specific for the class I MHC protein. CD8 is also one of the M1 pro-inflammatory markers. To assess CD8 levels, BV-2 ​cells were exposed to 100 ​ng/ml of LPS with or without CB86 and CB204 (Fig. 6). The levels of CD8 in LPS group were dramatically increased whereas in LPS ​+ ​CB86, the increment of CD8 levels was inhibited. In LPS ​+ ​CB204, the increment was inhibited but not as good as with CB86. As shown in Fig. 6, CB86 and CB204 did not have any effect on their own.

### 3.7 Cell metabolism assay

**Fig. 7:**
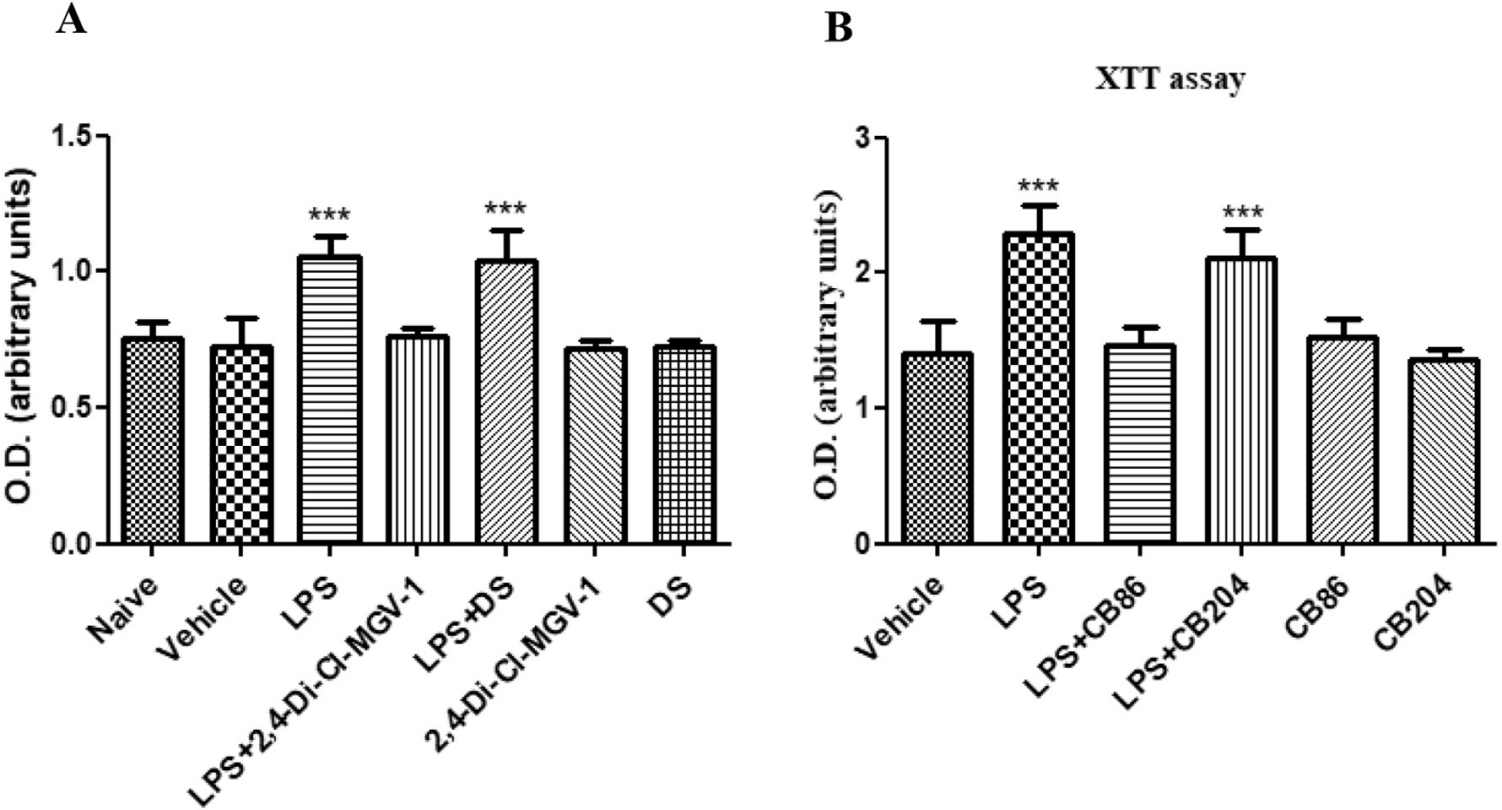
**Cell metabolism assay with XTT in BV-2 ​cells**. BV-2 ​cells were exposed to 100 ​ng/ml LPS for 24 ​h simultaneously with or without (A) 2,4-Di-Cl-MGV-1 and DS; (B) CB86 and CB204 (25 ​μM eacch). O.D. was recorded using an ELISA reader after XTT reaction and presented as Means ​± ​SD; 8 replicates in each group. ANOVA with Bonferroni’s post-hoc test was performed. ∗∗∗p ​< ​0.001 compared to all other groups.

Effects of the novel TSPO ligands (2,4-Di-Cl-MGV-1, CB86, CB204) and NSAID (DS) on cell metabolism, including mitochondrial metabolism, were compared in LPS-induced BV-2 ​cells (Fig. 7). The cells were exposed to LPS and treated simultaneously with or without 2,4-Di-Cl-MGV-1, CB86, CB204 and DS (25 ​μM each). No difference was observed between the naïve and vehicle control group as assayed by XTT. Metabolism in the LPS group was elevated significantly as compared to the control groups. DS did not affect significantly the metabolism the cells exposed to LPS. In contrast, co-treatment with 2,4-Di-Cl-MGV-1 of LPS restored the metabolism to control levels. In Fig. 7B, LPS ​+ ​CB86 levels were reduced to the control levels but CB204 did not have significant inhibitory effect on the response to LPS. Neither the TSPO ligands, nor DS showed effects on their own. ANOVA with Bonferroni’s post-hoc test was performed. ∗∗∗p ​< ​0.001 compared to all other groups.

### 3.8 Effect of TSPO ligands on microglial polarization to M2 pathway

**Fig. 8:**
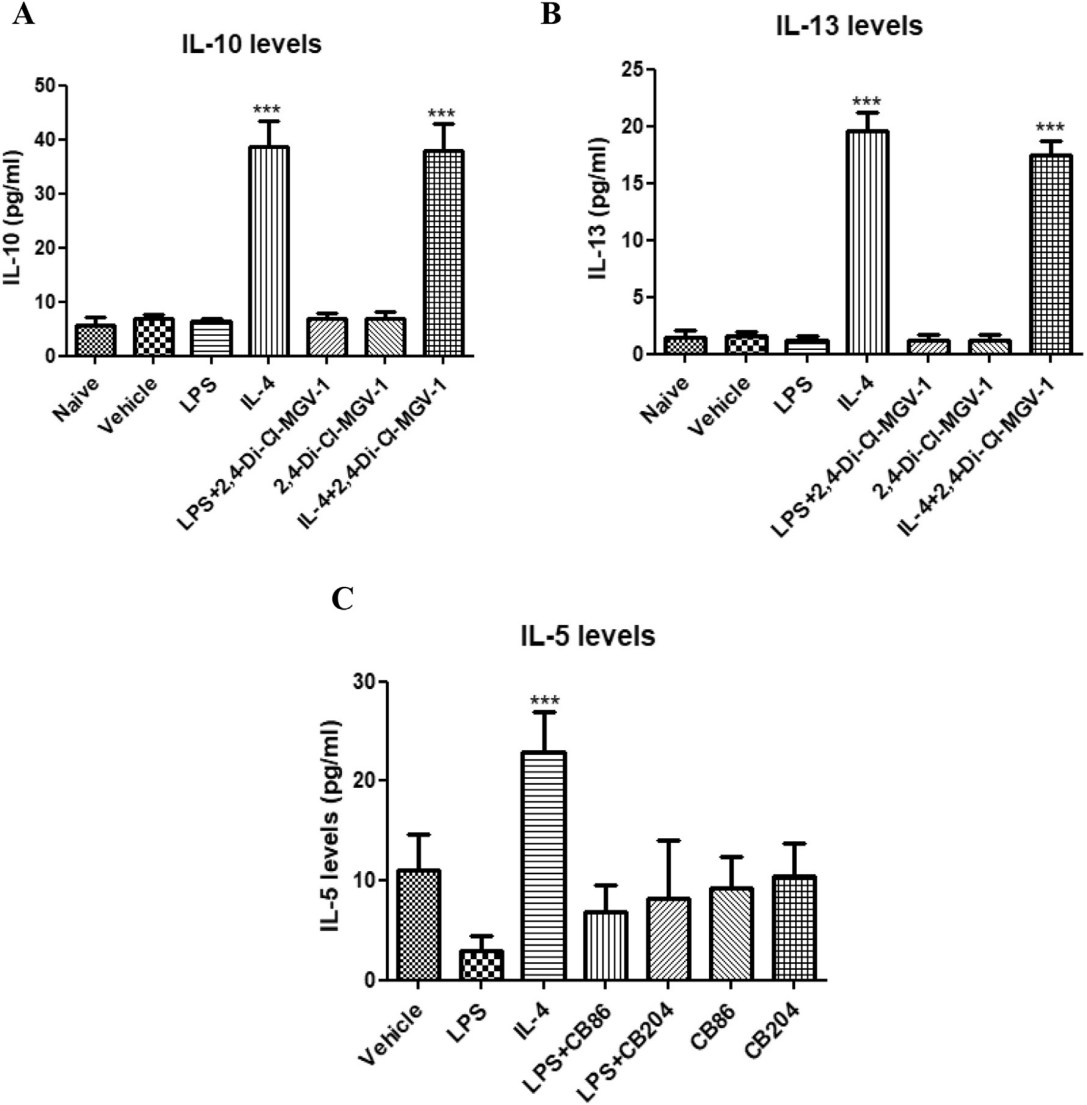
**Effect of TSPO ligands on IL-10, IL-13, and IL-5 levels in LPS- induced BV-2 ​cells (marker for M2 pathway).** BV-2 ​cells were exposed to 100 ​ng/ml LPS and 20 ​ng/ml of IL-4 for 24 ​h simultaneously with or without TSPO ligands (25 ​μM). (A) IL-10; (B) IL-13 concentrations (pg/ml) were calculated using a standard calibration curve and are presented as Means ​± ​SD; 4 replicates in each group. ANOVA with Bonferroni’s post-hoc test was performed. ∗∗∗p ​< ​0.001 compared to all other groups. (IL-10 and IL-13 presents marker for the M2 pathway).

This experiment was performed to assess the involvement of M2 pathway after following the treatment of 2,4-Di-Cl-MGV-1 along with LPS exposure. Exposure of the BV-2 ​cells to IL-4 was used as a positive control and LPS as negative control. IL-10 (Fig. 8 A) is one of the major markers for M2 pathway and it is not involved in M1 pathway. We also investigated another marker for M2 pathway namely, IL-13 (Fig. 8 B). Both IL-10 and IL-13 levels were stimulated by IL-4 and the TSPO ligand 2,4-Di-Cl-MGV-1 did not affect this response showing no effect at the M2 pathway. In case of Fig. 8C, LPS ​+ ​CB86 and LPS ​+ ​CB204 are not significantly different from control but LPS group is different from control. It seems that the two TSPO ligands CB86 and CB204 might affect M2 microglial polarization, but they did not have effect on their own.

## 4 Discussion

Our previous study has shown that TSPO ligands 2-Cl-MGV-1 and MGV-1 can counteract with microglial activation and have anti-inflammatory effects (Monga et al., 2019). In the current study, we showed that the novel TSPO ligand 2,4-Di-Cl-MGV-1 has better anti-inflammatory activity with regard to the inhibitory effect at the NF-κB p65. In the present study, we have shown anti-inflammatory effects of recently developed other two TSPO ligands CB86 and CB204 as well. These two ligands showed an inhibitory effect with regard to nitrite, TNF-α, CD-8, and cell metabolism. As mentioned, 2-Cl-MGV-1 and MGV-1 inhibited NF-κB p65 (pS536) by 64% and 57%, respectively (Monga et al., 2019); whereas inhibition with 2,4-Di-Cl-MGV-1 is around 80% at the same concentration (25 ​μM). This superiority of 2,4-Di-Cl-MGV-1 may be related to its better affinity to TSPO compared to the other two ligands (Table 1). Although, the affinity of PK 11195 is much better than all the three TSPO ligands (Table 1); yet our compounds are more potent inhibitors of microglial activation. This discrepancy may be related to the antagonistic properties of PK 11195 ​at the TSPO. However, it is still possible that the affinity to TSPO does not play a major role in the anti-inflammatory activities of the ligands. The major concern is the selectivity of the anti-inflammatory activity of the TSPO ligand 2,4-Di-Cl-MGV-1 to TSPO, particularly at the high concentration used (i.e. 1 -100 ​μM). For the future studies, it would be interestingly to assess the anti-inflammatory effects in TSPO knockout animals or knockout BV2 cell line in an attempt to determine the selectivity of 2,4-Di-Cl-MGV-1 to TSPO. However, the data on the effect of a low ligand concentration (i.e. 1 ​μM) (Fig. 1) support the putative role of TSPO in the neuroinflammation suppression achieved by 2,4-Di-Cl-NGV-1. We are planning in the future to perform the same experiments with TSPO knockout BV-2 ​cell line for the clarification of the role of TSPO in microglial activation or the possibility of an off-target effect. Activation of microglia is associated with release of pro-inflammatory cytokines that amplify the inflammatory response by activating and recruiting immune cells. In addition, microglia can release potent neurotoxins, such as TNF-α and others, which may cause neuronal damage. There are several neurodegenerative diseases in which microglial activation may play a significant role due to neurotoxic effects (Raine; Dickson et al., 1993; Banati et al., 1993; Nguyen et al., 2004). Microglial activation is associated also with upregulation of TLRs, independent of the type of challenge (McGeer et al., 1988; Bilbo, 2010). Activation of M1 microglia leads to over-synthesis and release of the pro-inflammatory mediators TNF-α, IL-1β, IL-6, IFN-γ, NO, iNOS, and COX-2 (Moehle and West, 2015) whereas, M2 polarized microglia produce anti-inflammatory markers such as IL-4, IL-10, and IL-13. (ChÃ¡vez-GalÃ¡n et al., 2015).

In a recent study, we have shown that the TSPO ligands 2-Cl-MGV-1 and MGV-1 counteract with microglial activation including the release of pro-inflammatory cytokines (Monga et al., 2019). We related this anti-inflammatory activity to an inhibitory affect at the NF- κB p65 complex (Da Pozzo et al., 2019). In the present study, the novel TSPO ligand 2,4-Di-Cl-MGV-1 was found to possess marked anti-inflammatory properties which may be better than the previous two TSPO ligands (2-Cl-MGV-1 and MGV-1). This new compound seems to be a potent suppressor of microglial activation as it significantly reduced the release of NO, IL-6, IL-1β, IFN-γ, and TNF-α by 91–98% in BV-2 ​cell line. These results were achieved by exposure to LPS (100 ​ng/ml) and 2,4-Di-Cl-MGV-1 treatment simultaneously (at the concentration of 25 ​μM). The anti-inflammatory activity of the ligand was much higher than that of the NSAID (DS). According to our previous study with 2-Cl-MGV-1 and MGV-1 (Monga et al., 2019), the induction of microglial activation was prevented by 92% whereas the new TSPO ligand 2,4-Di-Cl-MGV-1 suppressed microglial activation with a slightly better magnitude (up to 98%).

Attenuation of M1 phenotype while polarizing M2 phenotype is suggested to be a potential approach for the treatment of neuroinflammatory disorders (Hu et al., 2015). Just like our previous TSPO ligands 2-Cl-MGV-1 and MGV-1, the novel ligand 2,4-Di-Cl-MGV-1 also blocks the M1 pathway without polarizing the phenotype into M2 (Monga et al., 2019). The lack of activation of M2 phenotype may be an advantage since stimulation of this pathway is associated with hypersensitivity, allergy and tumor progression (Tham et al., 2014; Gharib et al., 2019). 2,4-Di-Cl-MGV-1 was found to be able to suppress M1 phenotype but did not promote polarization of microglia to the M2 phenotype as described previously with macrophages treatment to antibiotics (Burger et al., 2009; Kieseier, 2011; Huang et al., 2017). NF-κB has been implicated in the pathogenesis of several inflammatory diseases, such as rheumatoid arthritis, inflammatory bowel disease, multiple sclerosis, systemic lupus erythematosus, type I diabetes, chronic obstructive pulmonary disease and asthma. NF-κB is a protein complex that controls transcription of DNA, cytokine production and cell survival and is involved in cellular responses to stress, cytokines and free radicals and bacterial or viral antigens (Pai and Thomas, 2008; Vainshtein et al., 2015; Veenman et al., 2016; Perkins, 2007). In response to different cellular stimuli, NF-κB plays a complex role in different immune cell types and in different diseases states. In our study, 2,4-Di-Cl-MGV-1 inhibited the NF-κB p65 almost completely (to the control range). Interestingly, while the response at NF- κB p65 (pS536) was suppressed by 64% and 57% by 2-Cl-MGV-1 and MGV-1, respectively (Monga et al., 2019); CB86 has a significant inhibitory effect similar to that of MGV-1, 2-Cl-MGV-1 and 2,4-Di-Cl-MGV-1. Interestingly, CB86 is a TSPO ligand with very high affinity towards TSPO, even higher than PK 11195 (Denora et al., 2017; Iacobazzi et al., 2017). Thus, these ligands seem to be promising candidates for the treatment of central and peripheral inflammatory diseases.

## 5 Conclusion

In conclusion, our current study shows that the TSPO ligands 2,4-Di-Cl-MGV-1 and CB86 are efficacious in attenuating the activation of microglia and the corresponding release of pro-inflammatory cytokines. This anti-neuroinflammatory activity of the TSPO ligands is reflected by the prevention of the production and release of several inflammatory markers including NO, pro-inflammatory cytokines (IL-1β, TNF-α, IFN-γ, and IL-6), NF-Κb p65 (pS536), CD8 and COX-2. Even the 1 ​μM of 2,4-Di-Cl-MGV-1 significantly inhibited the NO production (Fig. 1). This marked anti-inflammatory activity may be relevant to neuroinflammatory diseases. Apriori, based on previous studies (Vainshtein et al., 2015), affinity may be a predictive factor for the efficacy of TSPO ligands regarding counteraction of harmful inflammatory responses. In particular, low affinity seems to be beneficial regarding anti-inflammatory effects. To our surprise, both the high affinity TSPO ligand, CB86 and relatively low affinity ligand 2,4-Di-Cl-MGV-1were both efficacious in counteracting inflammatory responses. Also surprising, another TSPO ligand CB204, with a very similar affinity compared to 2,4-Di-Cl-MGV-1 was quite ineffective in counteracting inflammatory responses. Thus, it appears that affinity is not a major factor in determining the effectiveness of TSPO ligands in the treatment of inflammatory responses. Regarding structure, comparative studies have shown that the compound of the family of CB86 and CB204 and the family of 2,4-Di-Cl-MGV-1, 2-Cl-MGV-1 and MGV-1 have quite different structures (Veenman et al., 2016). Nonetheless, the ligands of these two families have very similar anti-inflammatory effects. The relevance of these findings to in vivo microglial activation in the brain and spinal cord as well as to macrophage activation deserves further investigation.

## Funding

This study was supported by The 10.13039/501100003977Israel Science Foundation (Moshe Gavish, 1931/14).

## Authors contribution

S.M. designed, performed the experiments and wrote the first draft of the manuscript. This project was supervised by M.G., R.N., and A.W. Methodology and data curation, S.M. and M.G.; writing—review and editing, M.G., A.W., N.D., V.L. and M.F. The TSPO ligands BC86 and CB204 were synthesized by N.D. and V.L. The research was conducted in the laboratory of M.G. The 2,4-Di-Cl-MGV-1 TSPO ligand was synthesized in the Laboratory of I.M by S.S. All authors contributed to the preparation of the final version of the manuscript.

## Declaration of competing interest

The authors declare no conflict of interest.
